# Therapeutic Efficacy of Autologous Platelet Concentrate Injection on Macular Holes with High Myopia, Large Macular Holes, or Recurrent Macular Holes: A Multicenter Randomized Controlled Trial

**DOI:** 10.3390/jcm10122727

**Published:** 2021-06-21

**Authors:** Mirinae Kim, Jae-Yon Won, Seung-Yong Choi, Minhee Kim, Ho Ra, Donghyun Jee, Jin-Woo Kwon, Kui-Dong Kang, Young-Jung Roh, Young-Gun Park, Seungbum Kang, Jeong-Ah Shin, Hyeon-Woo Yim, Young-Hoon Park

**Affiliations:** 1 Department of Ophthalmology and Visual Science, Seoul St. Mary’s Hospital, College of Medicine, The Catholic University of Korea, Seoul 06591, Korea; dreamf7@naver.com (M.K.); cuteyg2000@gmail.com (Y.-G.P.); 2 Department of Ophthalmology and Visual Science, Eunpyeong St. Mary’s Hospital, College of Medicine, The Catholic University of Korea, Seoul 03312, Korea; jaywon24@naver.com (J.-Y.W.); john0730@hanmail.net (S.K.); 3 Department of Ophthalmology, Dankook University Hospital, Dankook University College of Medicine, Cheonan 31116, Korea; malisim@naver.com; 4 Department of Ophthalmology and Visual Science, Yeoido St. Mary’s Hospital, College of Medicine, The Catholic University of Korea, Seoul 07345, Korea; chriszz@naver.com (M.K.); youngjungroh@daum.net (Y.-J.R.); 5 Department of Ophthalmology and Visual Science, Bucheon St. Mary’s Hospital, College of Medicine, The Catholic University of Korea, Bucheon 14647, Korea; raho@catholic.ac.kr; 6 Department of Ophthalmology and Visual Science, St. Vincent’s Hospital, College of Medicine, The Catholic University of Korea, Suwon 16247, Korea; donghyunjee@catholic.ac.kr (D.J.); krnjs99@hanmail.net (J.-W.K.); 7 Department of Ophthalmology and Visual Science, Incheon St. Mary’s Hospital, College of Medicine, The Catholic University of Korea, Incheon 21431, Korea; kuidongkang@gmail.com; 8 Department of Ophthalmology and Visual Science, Daejeon St. Mary’s Hospital, College of Medicine, The Catholic University of Korea, Daejeon 34943, Korea; nole@catholic.ac.kr; 9 Department of Preventive Medicine, College of Medicine, The Catholic University of Korea, Seoul 06591, Korea; y1693@catholic.ac.kr

**Keywords:** autologous platelet concentration, macular hole, pars plana vitrectomy

## Abstract

We aimed to evaluate the anatomical and functional outcomes of pars-plana vitrectomy (PPV) with or without autologous platelet concentrate (APC) injection in patients with recurrent macular holes (MHs), large MHs, or MHs with high myopia. This multicenter, prospective, interventional randomized controlled trial was conducted from March 2017 to April 2020. Participants were randomly allocated to a PPV group or a PPV+APC group. All participants underwent standard 25-gauge PPV, and eyes in the PPV+APC group underwent PPV with intravitreal APC injection before air-gas exchange. A total of 117 patients were enrolled (PPV group: *n* = 59, PPV+APC group: *n* = 58). Hole closure was achieved in 47 participants (79.7%) in the PPV group and 52 participants (89.7%) in the PPV+APC group. There were no between-group differences in the anatomical closure rate or functional outcomes including best-corrected visual acuity, metamorphopsia, pattern-reversal visual evoked potential, or Visual Function Questionnaire-25 score. The use of APC injection does not improve the anatomical and functional outcomes of surgery for large MHs, recurrent MHs, or MHs with high myopia. The adjunctive use of APC can be considered in selected cases because it is not inferior to conventional MH surgery, is relatively simple to perform, and is not affected by the surgeon’s skill.

## 1. Introduction

Idiopathic macular hole (MH) was first described by Johnson and Gass in 1988 [1]. It is defined as a full-thickness anatomical defect of the neurosensory retina in the fovea. It usually occurs in the sixth and seventh decades of life, and its prevalence ranges from 0.2% to 0.7% [2,3,4,5]. The current gold standard for the treatment of MH is surgical intervention with pars-plana vitrectomy (PPV) and induction of posterior vitreous detachment with or without internal limiting membrane (ILM) peeling and gas tamponade [3,6]. Owing to recent advances in the surgical technique, the anatomical success rates of primary surgery have been reported as 85–90% [6,7,8]. However, some patients with MH have unsatisfactory outcomes after primary surgery, particularly those with large MHs with a diameter of 400 μm or greater [9,10,11,12], persistent or recurrent MHs after primary surgery [13,14], and MHs with high myopia [15,16,17,18].

Although conventional PPV is the mainstay treatment for MH, several additional techniques have been introduced to improve the anatomical and functional outcomes of surgery, including inverted ILM flap [11,19], lens capsular flap transplantation [20], tapping of the MH edges [21], and autologous serum injection [22,23].

Platelet extracts are known to contain many growth factors and have been shown to promote wound healing. Autologous platelet concentrate (APC) is used in the fields of plastic, orthopedic, cardiovascular, and maxillofacial surgery [24,25,26,27,28]. It has been reported to be useful for the treatment of MH, with an anatomical success rate of 90–98% [29,30,31,32]. The potential benefit of APC in MH surgery is especially important in patients with challenging cases of MHs, including large, persistent, and myopic MHs. However, no randomized controlled trial has been conducted on the adjunctive use of APC in MH surgery.

The aim of this study was to determine whether the adjunctive use of APC improves the anatomical and functional outcomes of surgery for large (≥400 μm) MHs, recurrent MHs, or MHs with high myopia.

## 2. Materials and Methods

### 2.1. Study Population

This multicenter, prospective, interventional randomized controlled trial was conducted across six study sites in South Korea from March 2017 to April 2020. This study adhered to the tenets of the Declaration of Helsinki, and the study protocol was approved by the institutional review board of the Catholic University of Korea. All participants provided written informed consent before enrollment. This clinical trial was registered with the Clinical Research Information Service on 23 February 2016 (registration number KCT0002686). This manuscript was prepared according to the Consolidated Standards of Reporting Trials guidelines.

We included subjects aged 20 years or older diagnosed with idiopathic full-thickness MH. Participants who met one of the following criteria were included: (1) full-thickness MH with a minimum diameter of 400 μm, (2) recurrent or persistent MH after primary surgery, or (3) MH with high myopia (refractive error ≥6.0 diopter or axial length >25.5 mm). The exclusion criteria included: (1) previous vitreoretinal surgery other than primary MH surgery, (2) traumatic MH, (3) glaucoma or other concomitant retinal diseases (e.g., age-related macular degeneration, retinal detachment, retinal vein occlusion, or diabetic retinopathy), (4) periocular inflammation or infection, (5) systemic inflammatory conditions (e.g., pneumonia, septicemia, hepatitis B, hepatitis C, syphilis, human T leukemia virus, or human immunodeficiency virus), (6) patients with hemodynamic instability or hematologic disorders who are unsuitable for APC treatment, (7) history of cancer within one year, (8) use of systemic anticoagulants, and (9) pregnancy.

At baseline, the participants’ detailed demographic and medical histories were collected and they underwent a thorough ophthalmic evaluation that included best-corrected visual acuity (BCVA) testing, slit-lamp microscopy, noncontact tonometry, dilated fundus examination, M-chart testing (Inami Co., Tokyo, Japan), fundus photography, spectral-domain optical coherence tomography (OCT; Spectralis HRA-OCT, Heidelberg Engineering, Heidelberg, Germany), and pattern-reversal visual evoked potential (PRVEP) testing. All participants were administered the National Eye Institute Visual Function Questionnaire-25 (VFQ-25), a self-report health and quality of life questionnaire with total scores ranging from 0 (worst health and quality of life) to 100 (best health and quality of life) [33].

### 2.2. Randomization and Masking

Participants were randomly allocated in a 1:1 ratio to the PPV group (control group) or the PPV+APC group (experimental group). Randomization was performed using block randomization by the Clinical Research Coordinating Center of the Catholic Medical Center. Randomization was performed on the day of surgery. The participants and clinical teams were masked, and only the surgeon was unmasked. The postoperative functional and anatomical outcomes were assessed by independent, masked retinal specialists, and not by the unmasked surgeon.

### 2.3. Intervention: Surgical Techniques

All participants underwent standard 25-gauge PPV with induction of posterior vitreous detachment, ILM peeling, and gas tamponade, with or without simultaneous cataract surgery. The ILM was peeled off by a minimum of one-disc diameter around the MH. In persistent MH after primary surgery, ILM peeling was conducted only in patients who had remnant ILM around the MH. Following fluid-air exchange, air-gas exchange with 14% perfluoropropane was performed. Eyes in the PPV+APC group underwent the same procedure with intravitreal APC injection before the air-gas exchange. The surgeon can decide to use inverted ILM flap to improve surgical outcomes. In this inverted ILM flap technique, an ILM remnant attached to the margins of the MH was inverted upside-down to cover the MH.

The APC was prepared according to previously described techniques [30,34]. Immediately preoperatively, 27 mL of venous blood was drawn from the patient’s antecubital vein into a syringe containing 3 mL of acid citrate dextrose formula A. The blood was gently transferred to a separation kit (Prosys PRS Bio kit; Prodizen Inc., Seoul, South Korea) in a sterile manner and centrifuged for three minutes at 3000 rpm at 20 °C. The supernatant buffy coat was extracted using a 1 mL sterile syringe, and 0.1 mL of the coat was injected into the eye through a 25-gauge port following the fluid-air exchange. The remaining APC underwent quantitative component analysis. Interleukin (IL)-1β, IL-4, IL-6, IL-10, angiopoietin, transforming growth factor (TGF)-β1, platelet-derived growth factor (PDGF), matrix metalloproteinase (MMP)-9, tumor necrosis factor (TNF)-α, and vascular endothelial growth factor (VEGF) were assayed with enzyme-linked immunosorbent assay.

Participants in the PPV group were instructed to lie in the prone position immediately after surgery and as much as possible for two weeks. Patients in the PPV+APC group were instructed to lie in the supine position for six hours after surgery, following which they were to lie in the prone position as much as possible for two weeks.

### 2.4. Outcome Measures

Postoperative OCT and BCVA were assessed at the one-week and one-, two-, four- and six-month follow-up visits. Postoperative metamorphopsia was assessed using an M-chart at the two- and six-month follow-up visits. Postoperative PRVEP testing and VFQ-25 were conducted at the six-month follow-up visit.

The primary outcome was anatomical closure of the MH at 24 weeks postoperatively as determined using spectral-domain OCT. Two independent retinal specialists, who were masked to treatment allocation, graded the anatomical closure. The secondary outcomes were a (1) change in MH size, (2) change in BCVA, (3) change in metamorphopsia as assessed using an M-chart, (4) change in PRVEP P100 latency, and (5) change in VFQ-25 score. Safety outcome measures included the incidence and severity of ocular and non-ocular adverse events (AEs). Intraocular pressure (IOP) was measured using noncontact tonometry at each visit, and an IOP spike of >21 mmHg was considered to be an ocular AE.

### 2.5. Statistical Analysis

Statistical analyses were performed on an intention-to-treat basis. Baseline variables were compared between the two groups using the chi-square test or the Wilcoxon rank sum test. Primary outcome data were analyzed using the chi-square test. Secondary outcome data were analyzed using the repeated-measures analysis of variance (ANOVA) with the Greenhouse–Geisser correction and Bonferroni’s post hoc analysis. We performed all statistical analyses using SAS (version 9.3; SAS Institute Inc., Cary, NC, USA). All *p* values were two-sided; *p* values < 0.05 were considered significant.

## 3. Results

### 3.1. Baseline Characteristics

**Figure 1:**
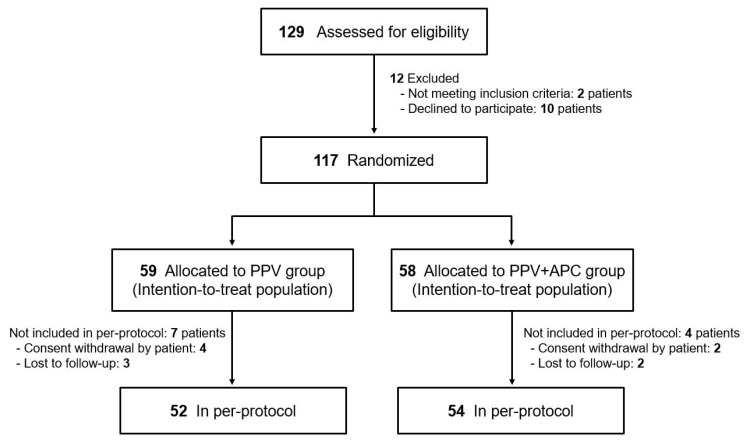
Flowchart of randomization and follow-up for the study (CONSORT flow diagram).

A total of 129 patients were screened for inclusion. Among them, 12 were excluded as they did not meet the inclusion criteria or declined to participate. The remaining participants were randomized to the PPV group (*n* = 59) or the PPV+APC group (*n* = 58). A total of 104 of the 117 randomized participants (88.9%) completed the 24-week follow-up and were included in the analysis (Figure 1).

The patient demographics and ocular characteristics at baseline were similar across the two groups (Table 1). The mean age of all the participants was 64.2 ± 7.7 years, and 83 (70.9%) participants were men. There were no between-group differences in axial length (*p* = 0.749), spherical equivalent (*p* = 0.788), intraocular pressure (*p* = 0.259), BCVA (*p* = 0.415), or lens status (*p* = 0.397).

### 3.2. Outcomes

#### 3.2.1. Primary Outcome

**Figure 2:**
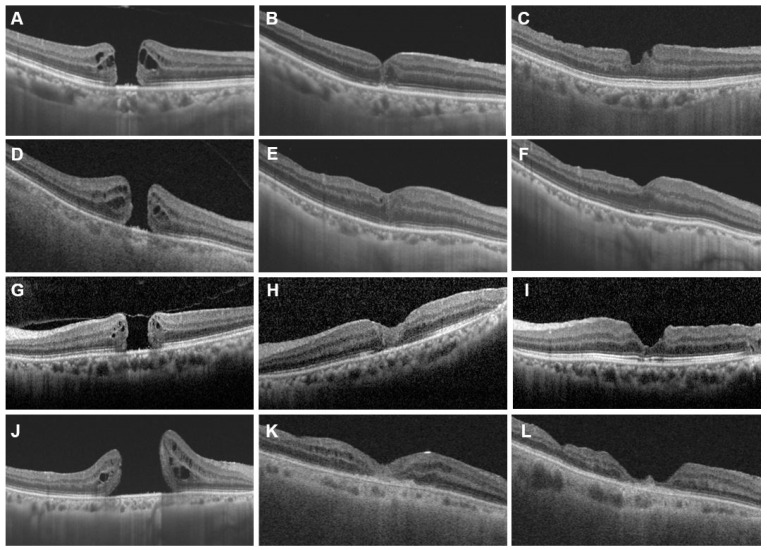
Representative cases showing preoperative and postoperative OCT images. Left row shows preoperative OCT, middle row shows OCT taken 1 month after surgery, and right row shows OCT taken 6 months after surgery. (**A**–**C**) OCT images of a 68-year-old woman with large MH (minimum hole diameter 531 um) (PPV+APC group). (**D**–**F**) OCT images of a 75-year-old man with MH with high myopia (axial length 25.56 um) (PPV+APC group). (**G**–**I**) OCT images of a 66-year-old woman with large MH (minimum hole diameter 564 um) (PPV group). Inverted ILM flap technique was used during surgery. (**J**–**L**) OCT images of a 69-year-old woman with large and persistent MH after primary surgery (PPV+APC group).

Data related to the primary outcome for both groups are shown in Table 2. Hole closure was achieved in 47 participants (79.7%) in the PPV group, and 52 participants (89.7%) in the PPV+APC group. There was no between-group difference in the anatomical closure rate (chi-square test, *p* = 0.134). Representative cases in each group are shown in Figure 2.

In the subgroup analysis, there was no difference in the anatomical closure rate. In large MH, hole closure was achieved in 80% in PPV group and 88.2% in PPV+APC group (*p* = 0.257). In myopic MH, hole closure was achieved in 84.6% in PPV group and 94.7% in PPV+APC group (*p* = 0.146). In recurrent/persistent MH, hole closure was achieved in 57.1% in PPV group and 60.0% in PPV+APC group (*p* = 0.921).

#### 3.2.2. Secondary Outcomes

Table 3 lists the change in MH size at each time point for both groups. There were no between-group differences in the minimum diameter (*p* = 0.964), baseline diameter (*p* = 0.949), or height of the MH (*p* = 0.530). Repeated-measures ANOVA revealed that the minimum diameter, baseline diameter, and height of the MH gradually decreased with time (*p* < 0.001, within-subject effects of time). Bonferroni post hoc analysis showed no statistically significant difference between the groups in minimum diameter, baseline diameter, or height of the MH at each time point. BCVA significantly improved over time (*p* < 0.001) but showed no statistically significant difference between the two groups (*p* = 0.130; Table 4).

Table 5 lists the change in metamorphopsia as assessed with an M chart. The M-chart score significantly improved over time (*p* < 0.001) but showed no statistically significant difference between the two groups (*p* = 0.762).

In the PPV group, the mean PRVEP P100 latency was 108.54 ± 1.72 ms at baseline and 109.48 ± 1.62 ms at 24 weeks postoperatively. In the PPV+APC group, the mean PRVEP P100 latency was 112.97 ± 2.33 ms at baseline and 110.10 ± 1.80 ms at 24 weeks postoperatively. There was no between-group difference in P100 latency (*p* = 0.313). The VFQ-25 total score was 72.8 ± 6.7 at baseline and 71.9 ± 5.5 at six months postoperatively in the PPV group, and it was 74.7 ± 5.3 at baseline and 73.0 ± 4.6 at six months postoperatively in the PPV+APC group. There was no between-group difference in self-reported visual function (*p* = 0.747).

### 3.3. Safety Outcomes

A total of 69 AEs were reported throughout the study period. Among them, 43 (62.3%) were reported in the PPV group and 26 (37.7%) in the PPV+APC group. The most common ocular AE was elevated IOP, which was seen in 34 patients (26.4%; 18 patients in PPV group and 16 patients in PPV+APC group). Except in one patient, the IOP was well controlled with IOP-lowering medications. One patient required additional glaucoma surgery. Other ocular AEs included cataract (five cases, 3.9%; two patients in PPV group and three patients in PPV+APC group), keratoconjunctivitis (five cases, 3.9%; three patients in PPV group and two patients in PPV+APC group), and newly detected MH in the non-study eye (one case, 0.8%; in PPV+APC group).

### 3.4. Component Analysis

Analysis of 56 patients from the PPV+APC group showed that APC contained 27.9 ± 27.1 pg/mL of IL-1β, 10.2 ± 9.2 pg/mL of IL-4, 35.7 ± 58.8 pg/mL of IL-6, 4.5 ± 4.1 pg/mL of IL-10, 11.5 ± 66.7 ng/mL of angiopoietin, 8.1 ± 5.9 ng/mL of TGF-β1, 2.5 ± 2.1 ng/mL of PDGF, 16.7 ± 16.1 ng/mL of MMP-9, 15.5 ± 18.7 ng/mL of TNF-α, and 29.3 ± 41.8 ng/mL of VEGF. There were no significant differences in the concentrations of these factors between the anatomical success group and the anatomical failure group (all *p* > 0.05).

## 4. Discussion

We think that this is the first randomized controlled trial to evaluate the benefit of adjunctive intravitreal APC injection during surgery for large (≥400 μm) MHs, recurrent MHs, or MHs with high myopia. We noticed a trend toward a higher anatomical success rate in the PPV+APC group. However, this difference was not statistically significant. Conclusively, the use of APC does not seem to improve the anatomical and functional outcome of surgery.

In general, the overall anatomical hole closure rates of large or recurrent MHs or MHs with high myopia are lower than those of other types of MHs. The anatomical closure rate of large MHs (>400 μm) was 56–87.5% [9,35,36]. Primary surgery for MHs with high myopia showed an anatomical success rate of 60–77% [16,17]. To improve the success rate of surgery, several surgical techniques including inverted ILM flap, lens capsular flap transplantation, and APC injection have been adopted.

APC has been widely used to accelerate the healing process. The α-granules of platelets contain abundant growth factors including PDGF, VEGF, TGF-β, insulin-like growth factor, and fibroblast growth factor [37,38,39]. The main growth factors observed in APC in our study were TGF-β1, VEGF, and PDGF. The use of APC also helps to avoid immune incompatibility as it is prepared from autologous blood [40,41]. Following surgery and gas tamponade, MH closure is mediated through the proliferation of glial cells. The role of the growth factors abundant in APC on MH closure has been extensively studied. In their prospective pilot study, Gaudric et al. reported that adjunctive APC injection during PPV significantly improved the anatomical and functional outcomes of patients with MH [29]. Paques et al. [30] also reported a greater anatomical success rate of MH surgery in the APC group (98%) than in the control group (85%).

In this study, the rates of intraoperative and postoperative complications were similar to those of conventional surgery. Postoperative IOP elevation was most commonly observed (26.4% of patients), but was well-controlled with medications. No APC-associated endophthalmitis or proliferative vitreoretinopathy occurred in either group. Consequently, adjunctive APC injection during PPV can be considered a safe procedure.

Despite the usefulness of APC injection, we did not observe a significant between-group difference in the anatomical closure rate. This may be because we used the inverted ILM flap technique in many patients, including those in the control group. In this study, inverted ILM flap technique during the PPV was conducted in 30 patients (25.6%; 25 patients in the PPV group and 5 patients in the PPV+APC group). The use of the inverted ILM flap technique might significantly improve the outcome of MH surgery. This might be a limitation of our study. However, it is important to note that only experienced surgeons can perform the inverted ILM flap technique. The surgeon’s comfort and experience with a particular technique should be considered during the selection of surgical technique. Consequently, despite there being no difference in primary outcome, we think that APC injection is advantageous as the preparation and manipulation of APC does not require surgeon’s experience.

Our study has several limitations. First, the use of additional surgical techniques including inverted ILM flap was permitted based on the surgeon’s decision, and the surgical techniques were not standardized. Second, there was no qualitative analysis on the OCT features before and after surgery. Despite these limitations, the strength of our study is that it is the first randomized controlled trial on the efficacy of APC injection during MH surgery for large MH, recurrent MH, and MH with high myopia.

In summary, we found no additional benefit of APC injection during MH surgery for large (≥400 μm) MHs, recurrent MHs, or MHs with high myopia. However, the adjunctive use of APC can be considered because it is not inferior to conventional MH surgery, is relatively simple to perform, and is not affected by the surgeon’s skill.

## Figures and Tables

**Table 1:** Demographics and baseline characteristics.

	PPV Group (*n* = 59)	PPV+APC Group (*n* = 58)	*p* Value
Age	63.4 ± 8.0	65.0 ± 7.4	0.371 *
Sex, male (%)	46 (78.0%)	37 (63.8%)	0.091 ^†^
Right side, *n* (%)	27 (45.8%)	32 (55.2%)	0.309 ^†^
Main symptom			0.091 ^†^
Decreased vision	41 (69.5%)	46 (79.3%)	
Metamorphopsia	9 (15.3%)	2 (3.4%)	
Both	9 (15.3%)	10 (17.2%)	
Systolic blood pressure, mmHg	122.3 ± 14.0	126.2 ± 13.7	0.228 *
Diastolic blood pressure, mmHg	74.3 ± 10.3	75.9 ± 8.7	0.531 *
Ocular examinations			
Axial length, mm	24.7 ± 2.6	24.8 ± 2.6	0.749 *
Spherical equivalent, diopter	−1.1 ± 2.7	−1.7 ± 4.2	0.788 *
Intraocular pressure, mmHg	14.8 ± 2.8	14.4 ± 3.4	0.259 *
BCVA (logMAR)	0.8 ± 0.5	0.8 ± 0.6	0.415 *
Lens status, *n* (%)			0.397 ^†^
Phakic	20 (33.9%)	22 (37.9%)	
Pseudophakic	39 (66.1%)	36 (62.1%)	

Data are expressed as mean ± standard deviation (95% confidence interval). * Wilcoxon rank sum test, ^†^ chi-square test. BCVA, best-corrected visual acuity; logMAR, logarithm of the minimum angle of resolution.

**Table 2:** Anatomical success rates of macular hole surgery in the two groups.

	PPV Group (*n* = 59)	PPV+APC Group (*n* = 58)	*p* Value
Status of macular hole			0.134
Closed	47 (79.7%)	52 (89.7%)	
Unclosed	12 (20.3%)	6 (10.3%)	

*p* value per the chi-square test.

**Table 3:** Comparison of macular hole size between the two groups.

		Baseline	1 Month	2 Months	4 Months	6 Months	*p* Value		
							Group (G)	Time (T)	G × T
Minimum Diameter	PPV	537.8 ± 26.4	78.0 ± 30.5	61.7 ± 29.1	51.8 ± 27.2	57.2 ± 30.2	0.964	<0.001	0.072
	PPV+APC	589.6 ± 29.2	57.3 ± 24.3	34.0 ± 20.1	39.1 ± 23.2	43.6 ± 22.1			
	Bonferroni post hoc test of the *p* value	0.192	0.595	0.432	0.724	0.714			
Base Diameter	PPV	967.0 ± 42.7	139.9 ± 50.4	110.8 ± 47.4	102.2 ± 48.3	101.9 ± 45.3	0.949	<0.001	0.050
	PPV+APC	1078.3 ± 49.9	115.2 ± 47.3	69.9 ± 40.9	74.9 ± 44.0	88.5 ± 44.5			
	Bonferroni post hoc test of the *p* value	0.094	0.721	0.514	0.676	0.833			
Height	PPV	438.3 ± 20.4	65.8 ± 24.1	50.9 ± 21.3	38.9 ± 17.2	36.1 ± 16.1	0.530	<0.001	0.276
	PPV+APC	464.4 ± 25.5	47.6 ± 19.2	26.9 ± 16.2	27.3 ± 16.1	38.5 ± 19.6			
	Bonferroni post hoc test of the *p* value	0.428	0.554	0.369	0.624	0.927			

Data are expressed as mean ± standard error. *p* value by repeated-measure ANOVA with the Greenhouse–Geisser correction (ε < 0.75), Bonferroni’s procedure to account for multiple testing.

**Table 4:** Comparison of mean best-corrected visual acuity (BCVA) between the two groups.

		Baseline	1 Month	2 Months	4 Months	6 Months	*p* Value		
							Group (G)	Time (T)	G × T
BCVA	PPV	0.84 ± 0.06	0.69 ± 0.06	0.69 ± 0.05	0.66 ± 0.05	0.68 ± 0.06	0.130	<0.001	0.256
	PPV+APC	0.85 ± 0.08	0.82 ± 0.07	0.77 ± 0.06	0.72 ± 0.05	0.68 ± 0.05			
	Bonferroni post hoc test of the *p* value	0.908	0.175	0.371	0.402	0.979			

Data are expressed as mean ± standard error. *p* value by repeated-measure ANOVA with the Greenhouse–Geisser correction (ε < 0.75), Bonferroni’s procedure to account for multiple testing.

**Table 5:** Comparison of metamorphopsia assessed with M-chart score between the two groups.

		Baseline	2 Months	6 Months	*p* Value		
					Group (G)	Time (T)	G × T
M-chart score	PPV	0.82 ± 0.06	0.64 ± 0.05	0.52 ± 0.05	0.762	<0.001	0.070
	PPV+APC	0.72 ± 0.06	0.70 ± 0.07	0.62 ± 0.07			
	Bonferroni post hoc test of the *p* value	0.296	0.463	0.268			

Data are expressed as mean ± standard error. *p* value by repeated-measure ANOVA with the Greenhouse–Geisser correction (ε < 0.75), Bonferroni’s procedure to account for multiple testing.

## Data Availability

Data available on request due to privacy/ethical restrictions. The data presented in this study are available on request from the corresponding author.
